# The C-terminus of Dpb2 is required for interaction with Pol2 and for cell viability

**DOI:** 10.1093/nar/gks880

**Published:** 2012-10-02

**Authors:** Isabelle Isoz, Ulf Persson, Kirill Volkov, Erik Johansson

**Affiliations:** Department of Medical Biochemistry and Biophysics, Umeå University, SE-90187 Umeå, Sweden

## Abstract

DNA polymerase ε (Pol ε) participates in the synthesis of the leading strand during DNA replication in *Saccharomyces cerevisiae*. Pol ε comprises four subunits: the catalytic subunit, Pol2, and three accessory subunits, Dpb2, Dpb3 and Dpb4. *DPB2* is an essential gene with unclear function. A genetic screen was performed in *S. cerevisiae* to isolate lethal mutations in *DPB2*. The *dpb2-200* allele carried two mutations within the last 13 codons of the open reading frame, one of which resulted in a six amino acid truncation. This truncated Dpb2 subunit was co-expressed with Pol2, Dpb3 and Dpb4 in *S. cerevisiae*, but this Dpb2 variant did not co-purify with the other Pol ε subunits. This resulted in the purification of a Pol2/Dpb3/Dpb4 complex that possessed high specific activity and high processivity and holoenzyme assays with PCNA, RFC and RPA on a single-primed circular template did not reveal any defects in replication efficiency. In conclusion, the lack of Dpb2 did not appear to have a negative effect on Pol ε activity. Thus, the C-terminal motif of Dpb2 that we have identified may instead be required for Dpb2 to fulfill an essential structural role at the replication origin or at the replication fork.

## INTRODUCTION

Three DNA polymerases, DNA polymerase α (Pol α), DNA polymerase δ (Pol δ) and DNA polymerase ε (Pol ε), participate at the replication fork during eukaryotic DNA replication (1,2). All three polymerases are multi-domain enzymes composed of several subunits encoded by separate genes (3). The functions of the smaller accessory subunits are not clear in all cases, but emerging evidence indicates that they are important for regulating the location and timing of DNA synthesis by the individual DNA polymerases (3). This regulation is critical because the replication fork is a dynamic structure that can allow the three different DNA polymerases to change places as their functions are required during DNA replication (1,2,4).

Pol α synthesizes the primer that is required at the origin and at the beginning of each Okazaki fragment on the lagging strand. The four subunits of Pol α are Pri1, Pri2, Pol1 and Pol12. The Pri1 subunit synthesizes a short RNA primer. This is followed by a switch to DNA synthesis by the Pol1 subunit that extends the RNA primer with a DNA primer that is up to 20 nucleotides long (5). The Pri2 and Pol12 subunits lack catalytic activity, but Pol12 is post-translationally modified during the cell cycle and in response to DNA damage (6). This suggests that at least Pol12 is functionally important for Pol α.

Increasing evidence suggests that Pol ε replicates the leading strand and Pol δ replicates the lagging strand during DNA replication (7–10). In *Saccharomyces cerevisiae*, Pol δ consists of three subunits, Pol3, Pol31 and Pol32. Pol3 is the catalytic subunit that synthesizes DNA and has an associated 3′–5′-exonuclease activity that can remove mis-incorporated nucleotides. *POL31* is an essential gene, but its *in vivo* function is unclear. *POL32* is not an essential gene in *S. cerevisiae* (11), but the Pol32 subunit plays important roles in the cell. The N-terminus of Pol32 interacts with Pol31 and the C-terminus interacts with PCNA, the processivity clamp, and Pol32 has been shown to be required for Pol δ to be fully processive in holoenzyme assays (11,12). Deletion of the *POL32* gene renders yeast strains cold sensitive and hydroxyurea sensitive, and *POL32* is required for ultraviolet (UV)-induced mutagenesis and break-induced repair (11,13,14). Recently, it was shown that Pol31 and Pol32 physically interact with Rev3 and may be subunits of the error-prone DNA polymerase ζ (15). Thus, Pol31 and Pol32 are functionally important even though they do not carry any of the catalytic sites of the Pol δ holoenzyme.

Pol ε is made up of four subunits, Pol2, Dpb2, Dpb3 and Dpb4 (16). Pol2 is the catalytic subunit with a DNA polymerization site and a 3′–5′-exonuclease site that proofreads the newly synthesized strand (17). *POL2* is an essential gene, but the deletion of the entire region that codes for the N-terminal domain of the protein that contains both the polymerase and proofreading activities did not result in lethality (18). The cells were viable, but did have a severe growth defect. However, a point mutation that inactivated the polymerase active site but kept this portion of the protein intact was lethal (19). The current model is that Pol δ is capable of rescuing leading strand synthesis at the replication fork when the 120 kD N-terminal fragment of Pol2 is missing, but cannot rescue synthesis in the presence of the inactivating point mutation. In contrast, the deletion of the region of the *POL2* gene encoding the C-terminus of Pol2 resulted in inviable cells. *DPB3* and *DPB4* are non-essential genes in *S. cerevisiae* (20,21). Dpb3 and Dpb4 form a complex that interacts with double-stranded DNA *in vitro* (22) and this may explain why Dpb3 and Dpb4 are important for the processivity of Pol ε (23,24). In addition, it was found that deletion of both *DPB3* and *DPB4* generated substrates for Pol ζ and increased the rate of mutagenesis (20,24,25). In contrast to the proofreading-deficient mutant of Pol ε (*pol2-4*), errors in DNA synthesis were not corrected by the mismatch repair system when *DPB3* and *DPB4* were deleted (24).

*DPB2* is an essential gene, but the role of Dpb2 in Pol ε activity is unclear (26). Temperature-sensitive (ts) alleles of *DPB2* have been isolated that cause a higher mutation rate *in vivo* (27,28). In two-hybrid assays, it has been shown that the increase in mutation rates conferred by different ts *dpb2* alleles is correlated with a reduced affinity between the mutant Dpb2 subunits and the C-terminus of Pol2 (29). Dpb2 is phosphorylated by the cyclin-dependent protein kinase (CDK) Cdc28 in late G_1_-phase, but the functional importance of this phosphorylation is unclear (30). A nuclear magnetic resonance solution structure of the N-terminal domain of Dpb2 has been solved, but the function of this domain remains unknown (31). Independent studies have suggested a role for Dpb2 during the initiation of DNA replication (32,33) and it has recently been shown that Dpb2 co-precipitates with the proteins that form the pre-loading complex (pre-LC) (33).

In the present study, we have isolated lethal *dpb2* alleles that have allowed us to investigate the role of Dpb2 in Pol ε function. We found that the Dpb2 protein expressed from one of these lethal alleles did not co-purify with Pol2, Dpb3 and Dpb4, and subsequent functional studies of the purified Pol ε complex that lacked Dpb2 showed that Pol ε does not depend on Dpb2 for the synthesis of new DNA. Thus, we propose that the essential function of Dpb2 is separate from the enzymatic activity of Pol ε in *S. cerevisiae* and that Dpb2 depends on a distinct domain at the very C-terminal end of the protein to physically interact with Pol2.

## MATERIALS AND METHODS

### Media and growth conditions

*Saccharomyces cerevisiae* strains were routinely grown in yeast complete media (YPD) that contained yeast extract (1%), peptone (2%) and glucose (2%). For transformation and selection, we used synthetic complete dropout—media (SC-media) that contained amino acid-free yeast nitrogen base, without amino acids and ammonium sulfate (0.34%), glucose (2%) and ammonium sulfate (0.5%) and was supplemented with the appropriate amino acids and nutrients as required. For selection of the kanamycin resistance gene (*KanR*) or the hygromycin B resistance gene (*hph*), G418 (200 µg/ml) or hygromycin B (200 µg/ml) was added to the YPD media, respectively. For plasmid shuffling, 0.1% 5-flouroorotic acid (5-FOA) was added to the SC media. Sporulation of *dpb2-200* heterozygote cells was performed on solid rich sporulation media that contained potassium acetate (1%), yeast extract (0.1%), glucose (0.05%) and agar (2%) and was supplemented with the appropriate amino acids and nutrients.

### Construction of a *DPB2*-deficient strain

Genomic DNA from *S*. *cerevisiae* strain E134 (*MATα ade5-1 lys2::InsEA14 trp1-289 his7-2 leu2-3,112 ura3-52*) (34) was used as a template for cloning the *DPB2* gene by polymerase chain reaction (PCR) with the primers p1 (5′-TATGGTACCCCGAAATCCGTTACATCAA-3′) and p2 (5′-TATGGATCCTAGAGGCACACAATCCTGAGAA-3′). The 2.7 kb PCR product was digested with KpnI and BamHI restriction enzymes and cloned into a pRS316 vector that contained the *URA3* gene sequence. The resulting vector was called pRS316-DPB2 and the *DPB2* gene was sequenced in both directions.

The chromosomal *DPB2* gene was deleted in E134 yeast cells that carried the pRS316-DPB2 plasmid by transforming the cells with a 1.7 kb PCR product that contained *DPB2*-flanking regions. This PCR product was amplified from pFA6a-KanMX6 (35) with primers p3 (5′-TCTATGTGTGAAATGTTTGGCTCTGGGAATGTTCTGCCTGTTAAAATTCAGCCTCCAGCTGAAGCTTCGTACGC-3′) and p4 (5′-CAGAGTACCAACACTACCGTGCTTGTTTAAGTGTTCTTATTTTGATGCATTATTTAGCATAGGCCACTAGTGGATCTG-3′). E134 cells were transformed with the PCR product, the integration was confirmed by PCR (Supplementary Figure S1), and the resulting strain was called E134-*dpb2Δ*.

### Isolation of alleles that carry lethal mutations in *DPB2*

The pRS316-DPB2 plasmid was digested with KpnI and BamHI and the *DPB2* fragment was cloned into a *TRP1*-containing pRS314 vector. The resulting vector was called pRS314-DPB2. Random mutagenesis was performed on the pRS314-DPB2 plasmid by treating it with hydroxylamine (36).The mutated plasmids were purified with a plasmid purification kit (Qiagen) to remove any traces of hydroxylamine before transforming the E134-*dpb2Δ* cells. Transformants were selected on SC-Ura-Trp plates and were replica plated onto SC-Trp plates containing 5-FOA to identify those transformants unable to proliferate in the absence of the plasmid with the wild-type *DPB2*. Plasmids from such transformants were isolated and the mutated *dpb2* alleles were subcloned into an untreated pRS314 vector. Lethality was confirmed by the same shuffling procedure. The vectors with lethal *dpb2* alleles were purified and sequenced.

### Construction of a diploid strain heterozygous for *dpb2-200* and tetrad analysis

*Saccharomyces cerevisiae* strains E134 and w1588-4c (*MATa leu2-3,112 trp1-1 can1-100 ura3-1 ade2-1 his3-11,15 RAD5+*) (37) were crossed to create a wild-type diploid strain homozygous for *DPB2* (*DPB2*/*DPB2*). To obtain the *dpb2-200* heterozygote (*dpb2-200*/*DPB2*), the *dpb2-200* allele was labeled with a hygromycin resistance gene, *hph*, and integrated into the genome of the diploid strain by homologous recombination. To do this, a DNA fragment containing *dpb2-200* was cut from the pRS314–dpb2-200 plasmid with KpnI and PstI endonucleases, blunt ended with T4 DNA polymerase and ligated into a SmaI-treated pAG32 vector. The resulting pAG32–dpb2-200 plasmid was used as a template to PCR amplify the *dpb2-200* fragment flanked by the *hph* gene. The amplification was performed with primers DPB2-6 (5′-AAAATTAAATGATGACCCACCG-3′) and p5 (5′-CAGAGTACCAACACTACCGTGCTTGTTTAAGTGTTCTTATTTTGATGCATTATTTAGCATAGGCCACTAGTGGATCTG-3′) to generate a PCR fragment containing the last third of the *dpb2-200* allele that included the mutation, the *hph* gene, and 60 bp of *DPB2* flanking sequences (Supplementary Figure S2). This PCR product was used to transform the diploid *DPB2*/*DPB2* strain. Transformants were selected on YPD + hygromycin B media, and the correct insertion of *dpb2-200* into the chromosome was verified by PCR using the primers described in Supplementary Figure S2 and by sequencing.

The *DPB2* homozygote and the *dpb2-200* heterozygote strains were grown on solid YPD media for 2 days before they were patched onto sporulation media and incubated for 5 days at 30°C. After the incubation, tetrads were dissected on YPD with a Singer MSM System 300 micromanipulator and the spores were incubated for 2 days at 30°C.

### Co-expression and purification of Pol2, mutated Dpb2, Dpb3 and Dpb4

The galactose-inducible over-expression vectors pJL1 (Pol2), pJL9-201 (Dpb2-201) and pJL5 (Dpb3 and Dpb4) were used to transform yeast strain pY116 cells. The resulting Pol ε complex was purified as previously described (23) with a comparable yield to wild-type Pol ε but lacking the Dpb2 subunit. The stoichiometry of Pol2:Dpb3:Dpb4 was estimated to be 1:1.1:1.4 after measuring the intensities of the Coomassie-stained protein bands on a 10% sodium dodecyl sulfate-polyacrylamide gel electrophoresis (SDS–PAGE). Overall, we found the biochemical properties of the Pol2/Dpb3/Dpb4 complex comparable to wild-type Pol ε.

A GST tag was inserted at the 5′-end of *POL2* in the plasmid pJL1, the 5′-end of *DPB2* in the plasmid pJL9 and the 5′-end of *dpb2-201* in the plasmid pJL9-201. The fusion proteins GST-Pol2, GST-Dpb2 and GST–dpb2-201 were over-expressed in PY116 cells together with the remaining wild-type subunits as described above. The harvested cells were lysed, fractionated by ammonium sulfate precipitation, loaded onto and eluted from a phosphocellulose column, and loaded onto a glutathione sepharose column as described previously (38). The glutathione sepharose column was washed with 20 column volumes of Buffer B (25 mM Hepes-NaOH pH 7.6, 10% glycerol, 1 mM ethylenediaminetetraacetic acid (EDTA), 0.5 mM ethyleneglycoltetraaceticacetic (EGTA), 0.005% Nonidet P-40, 1 mM dithiothreitol, 5 µM pepstatin A, 5 µM leupeptin and 5 mM NaHSO_3_). The bound protein was eluted with 20 mM glutathione (pH 8.0) in Buffer B. The eluted protein was colloidal Coomassie-stained after electrophoresis through a 10% SDS–PAGE (16).

### DNA polymerase assays

The specific activity of Pol ε was measured as previously described (16) and the primer extension assay was also performed as previously described (24). For the primer extension assay, a [γ-^32^P]ATP-labeled 50mer oligonucleotide was annealed to a pBluescript II SK(+) single-stranded DNA in a ratio of 1:1.5. For the DNA synthesis processivity assay, the substrate (14 nM) was incubated with the four-subunit wild-type Pol ε or the Pol2/Dpb3/Dpb4 complex as indicated in the corresponding figures. Reactions were performed in a mixture of 40 mM Tris–HCl pH 7.8, 1 mM dithiothreitol, 0.2 mg/ml bovine serum albumin, 8 mM MgCl_2_, 125 mM NaAc and 100 µM dNTPs. Each reaction was incubated at 30°C for 2 or 8 min and terminated with the addition of 15 μl of stop solution (80% formamide and 10 mM EDTA). The replication products were denatured at 95°C for 4 min and cooled on ice before electrophoresis through an 8% denaturing polyacrylamide gel. The gel was dried and placed on a phosphoimager screen (Fuji) and the intensity of the bands corresponding to different replication products was quantified with a Typhoon 9400 phosphoimager and the ImageQuant software package (GE Healthcare). The reaction conditions were empirically determined to meet single-hit kinetics conditions in which a polymerase molecule never re-associates with a previously extended primer (39).

Holoenzyme assays were performed in a standard 15 μl reaction that contained 40 mM Tris–HCl (pH 7.8), 1 mM dithiothreitol, 0.2 mg/ml bovine serum albumin, 8 mM MgAc_2_, 125 mM NaAc, 100 µM each of dGTP, dATP and dTTP, 50 µM dCTP, 1.5 µCi [α-^32^P] dCTP (Perkin Elmer), 70 fmol single-primed pBluescript II SK(+), 143 fmol DNA polymerase, 10.5 pmol RPA, 80 fmol RFC and 1.15 pmol PCNA. The reactions were incubated at 30°C for the times indicated in the figures. The reactions were terminated by adding 6 μl of stop solution (54 mM EDTA, 1% SDS, 0.135% Orange G, 0.027% xylene cyanol FF, 54% glycerol and 9 mM Tris–HCl pH 7.6). The samples were loaded onto a 0.8% alkaline agarose gel that contained 30 mM NaOH and 2 mM EDTA. The gels were run at 30 V for 16 h and neutralized with 0.1 M Tris–HCl pH 7.6. The gels were dried, placed on a phosphoimager screen (Fuji) and scanned with a Typhoon 9400 phosphoimager (GE Healthcare).

### Temperature sensitivity assay of the strains containing *dpb2-200* and *dpb2-201* alleles

E134-*dpb2Δ* cells that contained the pRS316-DPB2 plasmid were transformed with pRS314 vectors that carried the *dpb2-200*, *dpb2-201* or *DPB2* alleles, and selected on SC-Ura-Trp plates. The three different transformants were patched onto YPD plates and grown overnight at 30°C followed by replica plating onto SC-Ura-Trp and SC 5-FOA plates. The cells were grown at 16, 23, 30 and 37°C.

## RESULTS

### Isolation of lethal mutations in *DPB2*

**Figure 1.**
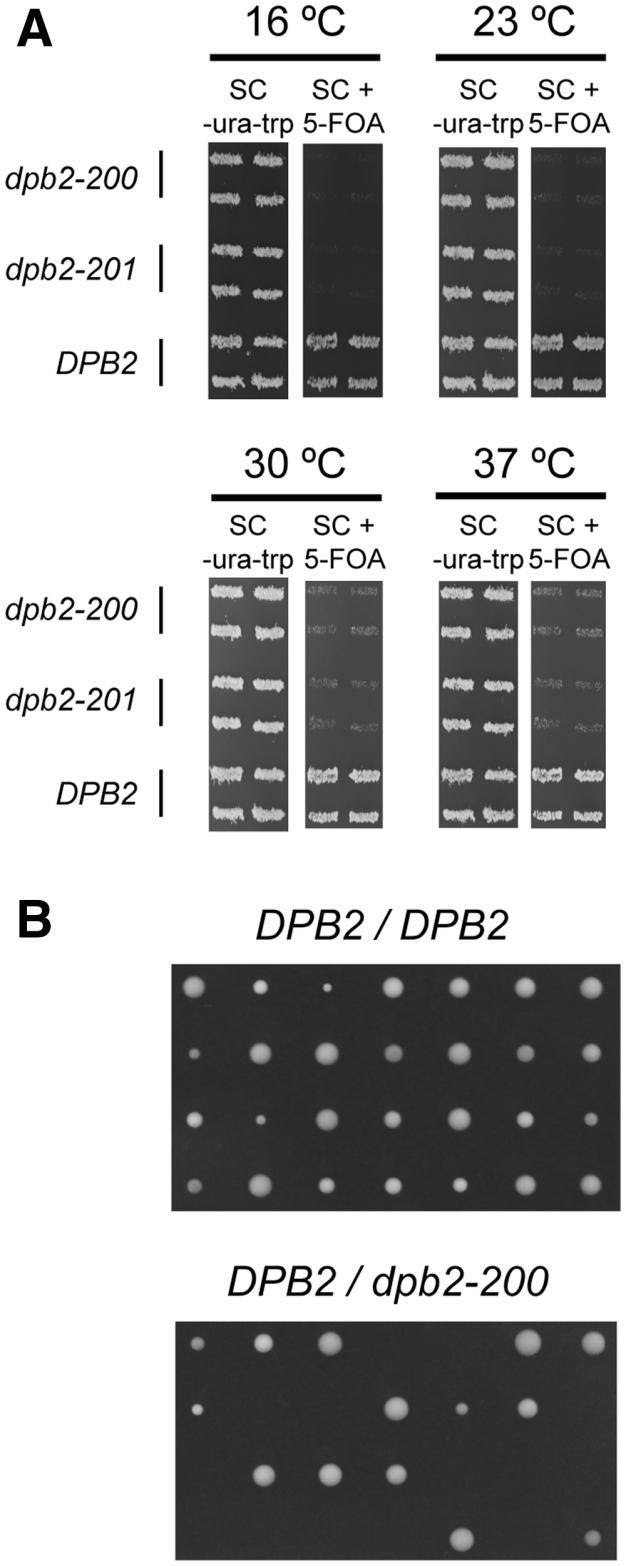
The isolated *dpb2-200* and *dpb-201* alleles cannot support growth. (**A**) The two alleles, *dpb2-200* and *dpb2-201*, were tested for lethality and temperature sensitivity in plasmid shuffling experiments in E134-*dpb2Δ* cells. 5-FOA was used for selection against the wild-type *URA3*-containing pRS316-DPB2 plasmid. The pRS314-DPB2 plasmid with the *TRP1* marker was the wild-type control that could grow on 5-FOA. Four clones of each transformant are presented. (**B**) Tetrad analysis of a *DPB2/DPB2* homozygote and a *DPB2/dpb2-200* heterozygote.

We isolated lethal mutations in the *DPB2* gene to examine the role of Dpb2 *in vivo* and its function in the Pol ε complex. *DPB2* is an essential gene, so to screen for lethal mutations we constructed two separate low copy number plasmids that carried the wild-type *DPB2* gene under the control of the wild-type *DPB2* promoter. One plasmid carried *TRP1* and the other carried *URA3* as selectable markers, and the plasmid with *TRP1* was mutagenized by treatment with hydroxylamine. Lethal mutations in the *DPB2* gene were identified by plasmid shuffling between the wild type and mutagenized *DPB2* genes in the E134-*dpb2Δ* yeast strain. That is, transformants carrying both the mutagenized plasmid and the *DPB2* wild-type plasmid were first selected on SC-Ura-Trp plates followed by replica plating onto SC −Trp + 5-FOA to identify those unable to proliferate in the absence of the plasmid with both the *URA3* gene and the wild-type *DPB2* gene. These cells carried a potentially lethal mutation in the hydroxylamine-treated *DPB2* gene. In the initial screen, 360 colonies were replica plated, and 11 of these were unable to grow on SC-Trp + 5-FOA. These 11 isolates were re-streaked and their inability to grow on 5-FOA was confirmed. The mutagenized plasmids were recovered from the yeast cells and the *DPB2* gene was sequenced in both directions. We identified three separate plasmids that carried mutations in *DPB2* and all three resulted in a truncated protein. Allele *dpb2-198* carried a nonsense codon that led to a truncation at residue 437 and allele *dpb2-199* carried three mutations (P592S, Q597stop and Q600stop) leading to a truncation at residue 597 (Table 1). One of the alleles, *dpb2-200,* carried two mutations located near the 3′-end of the *DPB2* gene. These two mutations changed a proline to serine at amino acid 680 (P680S) and a glutamine to a nonsense codon at position 687 (Q687stop). The *dpb2-200* allele was the most interesting allele because the two mutations were located within 13 residues of the C-terminus of the resulting protein and the truncation was only six residues. As the entire vector had been treated with hydroxylamine, we subcloned the *dpb2-200* allele into an untreated pRS314 vector to confirm that the lethal phenotype was not due to any additional mutations in the vector backbone. We also tested whether the *dpb2-200* allele was temperature sensitive and found that raising or lowering the temperature had no effect on viability (Figure 1A). Finally, we established a diploid strain that carried one *dpb2-200* allele and one wild-type *DPB2* allele. Tetrad analysis confirmed that *dpb2-200* was a lethal mutation because only two viable spores were found in each of seven asci examined (Figure 1B). This was not an integration effect caused by the hygromycin resistance gene because integration of *DPB2* using the same strategy gave four viable spores from each ascus (Supplementary Figures S2 and S3).

**Table 1.** Alterations in the amino acid sequence of the Dpb2 mutations described in this study

Dpb2	431-PPTLLI**W**QGS-440
Dpb2-198	431-PPTLLI * QGS-440
	
Dpb2	581-DIVPIDQLVK E**P**DQLP**Q**KV**Q** ETRKLVKTIL-610
Dpb2-199	581-DIVPIDQLVK E**S**DQLP* KV * ETRKLVKTIL-610
	
Dpb2	671-RRARYMEYV**P** SSKKTI**Q**EEI YI*-692
Dpb2-200	671-RRARYMEYV**S** SSKKTI * EEI YI*-692
	
Dpb2	671-RRARYMEYV**P** SSKKTI**Q**EEI YI*-692
Dpb2-201	671-RRARYMEYV**S** SSKKTI **-686

Amino acid substitutions and stop codons (*) are in bold.

### Lack of interaction between Dpb2-201 and Pol2, Dpb3 and Dpb4

**Figure 2.**
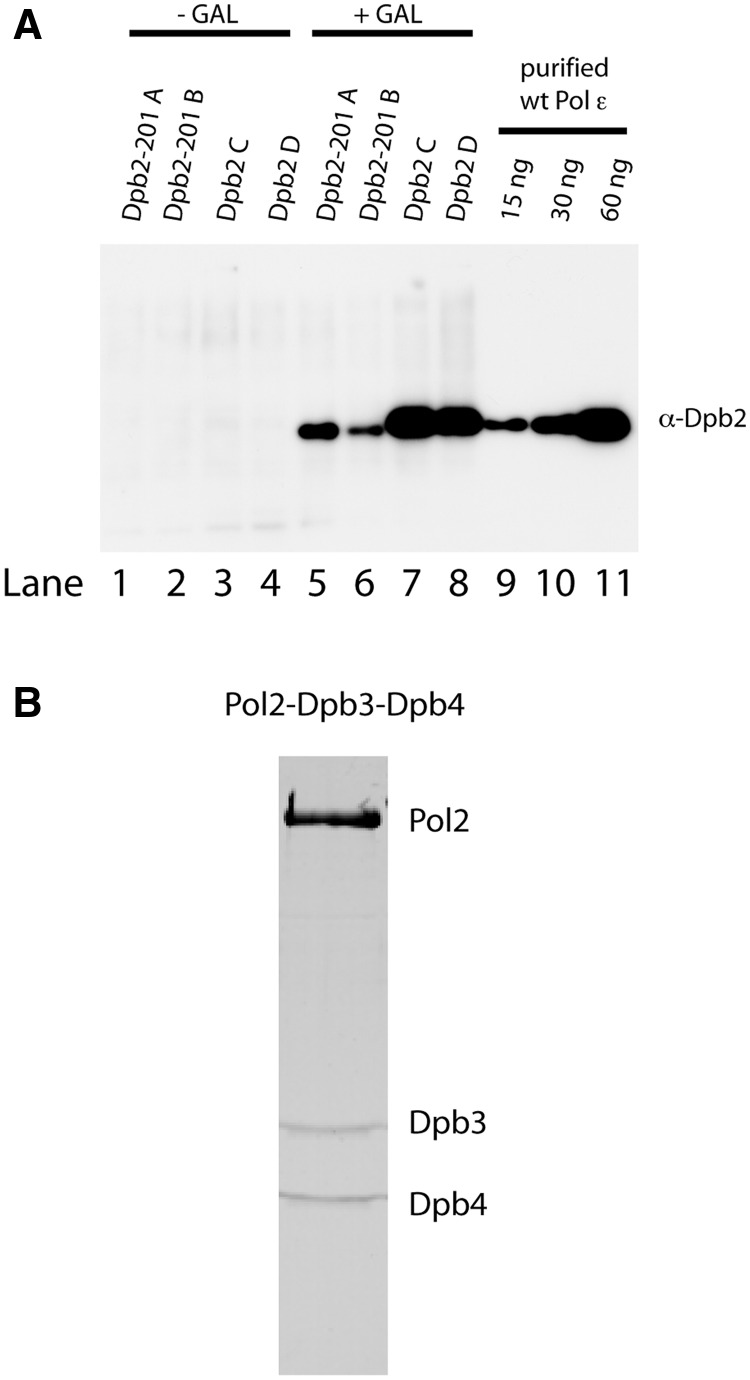
Co-expression of Dpb2-201 together with Pol2, Dpb3 and Dpb4. (**A**) Western-blot analysis of pY116 cells (16) that over-expressed Dpb2-201 or Dpb2 in the presence of galactose. A rabbit polyclonal antibody was used that recognizes Dpb2. Two isolates carrying either over-expression plasmids with *dpb2-201* or *DPB2* were analyzed. An equal amount of cells were loaded in each lane after boiling in a loading buffer with SDS, β-mercaptoethanol, bromophenol blue and glycerol. The first four lanes (1–4) are cells harvested before induction with galactose and the next four lanes (5–8) are cells harvested after induction with galactose. Lanes labeled A, B, C and D are the same cultures before and after induction, respectively. Fifteen nanograms, 30 ng and 60 ng of purified Pol ε were loaded as indicated in the last three lanes (9–11). (**B**) The purified Pol ε complex was missing the Dpb2 subunit when purified from cells with galactose-induced over-expression of Dpb2-201 (Figure 2A). The estimated subunit stoichiometry of the purified Pol2/Dpb3/Dpb4 complex was 1:1.1:1.4 based on a plot of the density of the Coomassie-stained bands.

To determine whether the lethal phenotype was due to a non-functional Pol ε, we created an allele called *dpb2-201* that was identical to *dpb2-200* except that the five codons after the Q687stop mutation were removed. This was done to minimize the risk of accidentally reading through the stop codon during translation. We first confirmed that the *dpb2-201* allele was lethal in the low copy vector pRS314–dpb2-201 (Figure 1A). Next, we cloned the *dpb2-201* allele into an over-expression vector under the control of the galactose-inducible promoter (plasmid pJL9-201) and co-expressed Dpb2-201 together with Pol2, Dpb3 and Dpb4. The Dpb2-201 protein was detected in a western blot of cell extracts obtained after induction with galactose (Figure 2A). Next, we applied a previously developed purification protocol for Pol ε that depends on ion-exchange chromatography and size-exclusion chromatography and found that Dpb2-201 did not co-purify with the other subunits (Figure 2B). This suggested that Dpb2-201 was unable to interact with the other Pol ε subunits.

**Figure 3.**
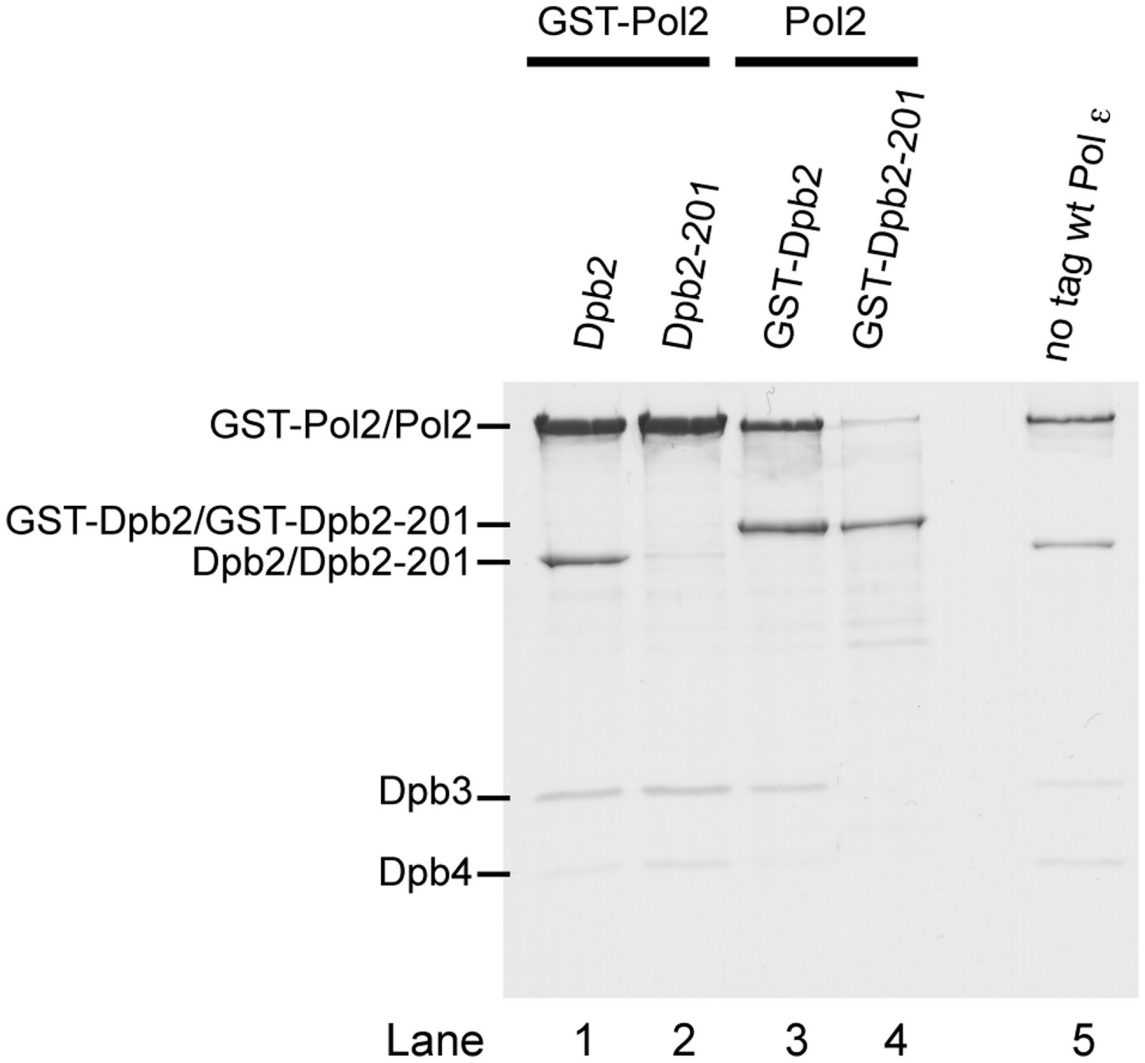
Pull-down experiment with GST-Pol2, GST-Dpb2 and GST–Dpb2-201. The positions of each subunit of Pol ε are indicated to the left. The amount of protein that was loaded in each lane was titrated such that an equal amount of GST-Pol2 was loaded in lanes 1 and 2, and equal amounts of GST-Dpb2 and GST–Dpb2-201 were loaded in lanes 3 and 4. The gel was colloidal Coomassie-stained (16).

To further test this hypothesis, we positioned a GST tag at the N-terminus of Pol2 in the over-expression system described above and asked whether over-expressed Dpb2 or Dpb2-201 would co-purify with GST-Pol2 on a glutathione-sepharose column. Only Dpb2, Dpb3 and Dpb4 co-purified with Pol ε over the affinity column (Figure 3, lanes 1 and 2), supporting our previous result that suggested that Dpb2-201 might have lost the ability to interact with Pol2 (Figure 2B). However, the western blots suggested that the Dpb2-201 protein was less abundant than wild-type Dpb2 when over-expressed in yeast (Figure 2A). This could potentially lead to a substoichiometric amount of Dpb2-201 in our Pol ε preparations and only give the impression that Dpb2-201 had lost its ability to interact with Pol2. Thus, we positioned a GST tag at the N-terminus of Dpb2 and Dpb2-201 to determine whether they would co-purify with Pol2 on a glutathione-sepharose column. Equal amounts of GST-Dpb2 and GST–Dpb2-201 were loaded in each lane. The amount of Pol2 co-purifying with GST–Dpb2-201 was significantly less compared with the GST-Dpb2 purification (Figure 3, lanes 3 and 4). Altogether, these results support a model in which Dpb2 depends on its C-terminus to interact with Pol2, but the small amount of Pol2 co-purifying with GST–Dpb2-201 suggests that there may exist a second, weak interaction surface. Furthermore, we observed a slight shift in the stoichiometry of Dpb3 and Dpb4 in the absence or presence of Dpb2 or GST-Dpb2.

### Biochemical characterization of the Pol2/Dpb3/Dpb4 complex

**Figure 4.**
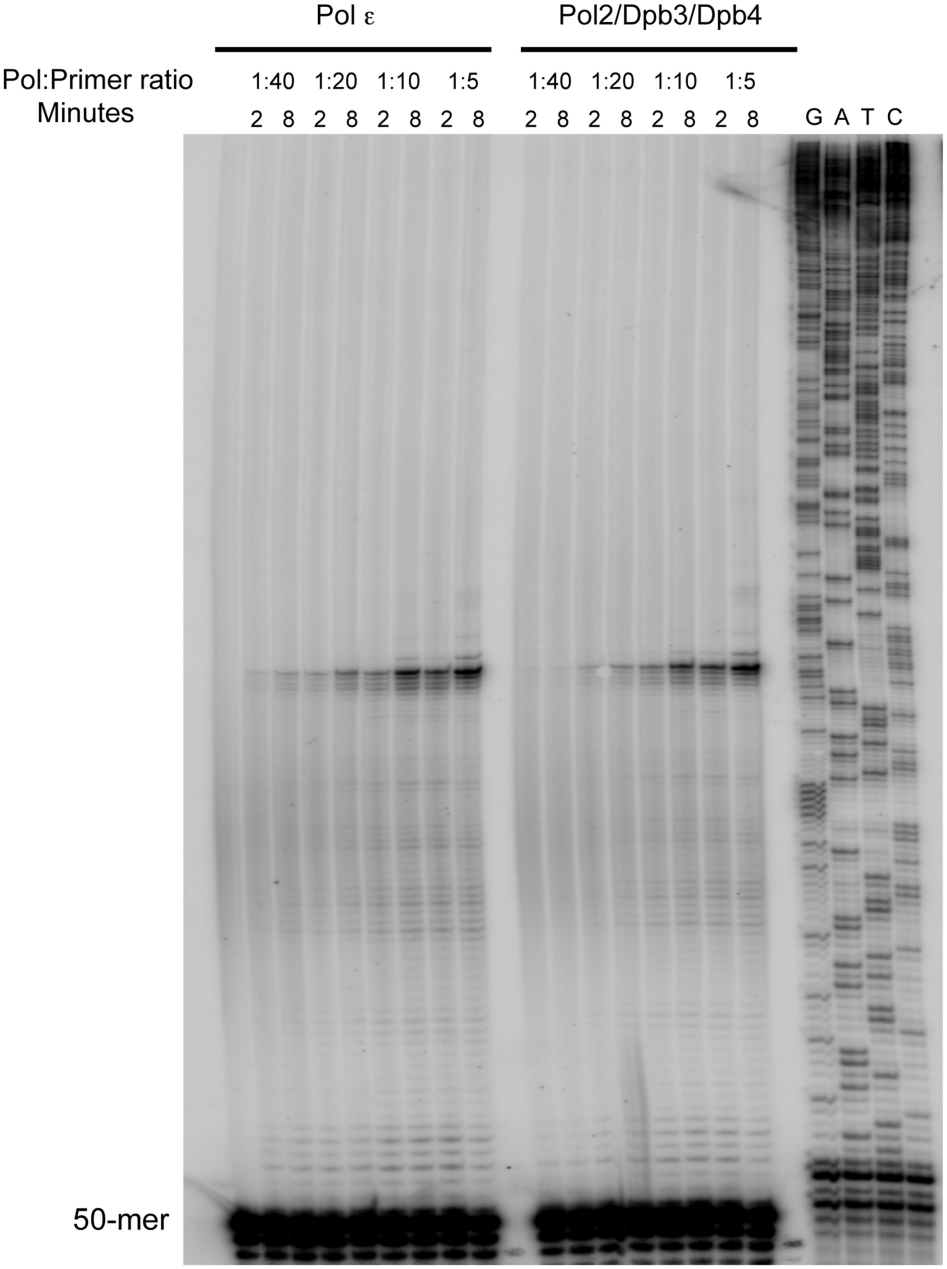
Primer extension assays with four-subunit Pol ε and the Pol2/Dpb3/Dpb4 complex. The experiments were performed with different ratios of enzyme to primer and template ([E]:[PT]). Results are shown after 2 and 8 min, as indicated. The termination probability was measured for each product as described previously (39). Sequencing reactions with the same primer and template used in the primer-extension assay were loaded to the right as a size marker.

**Figure 5.**
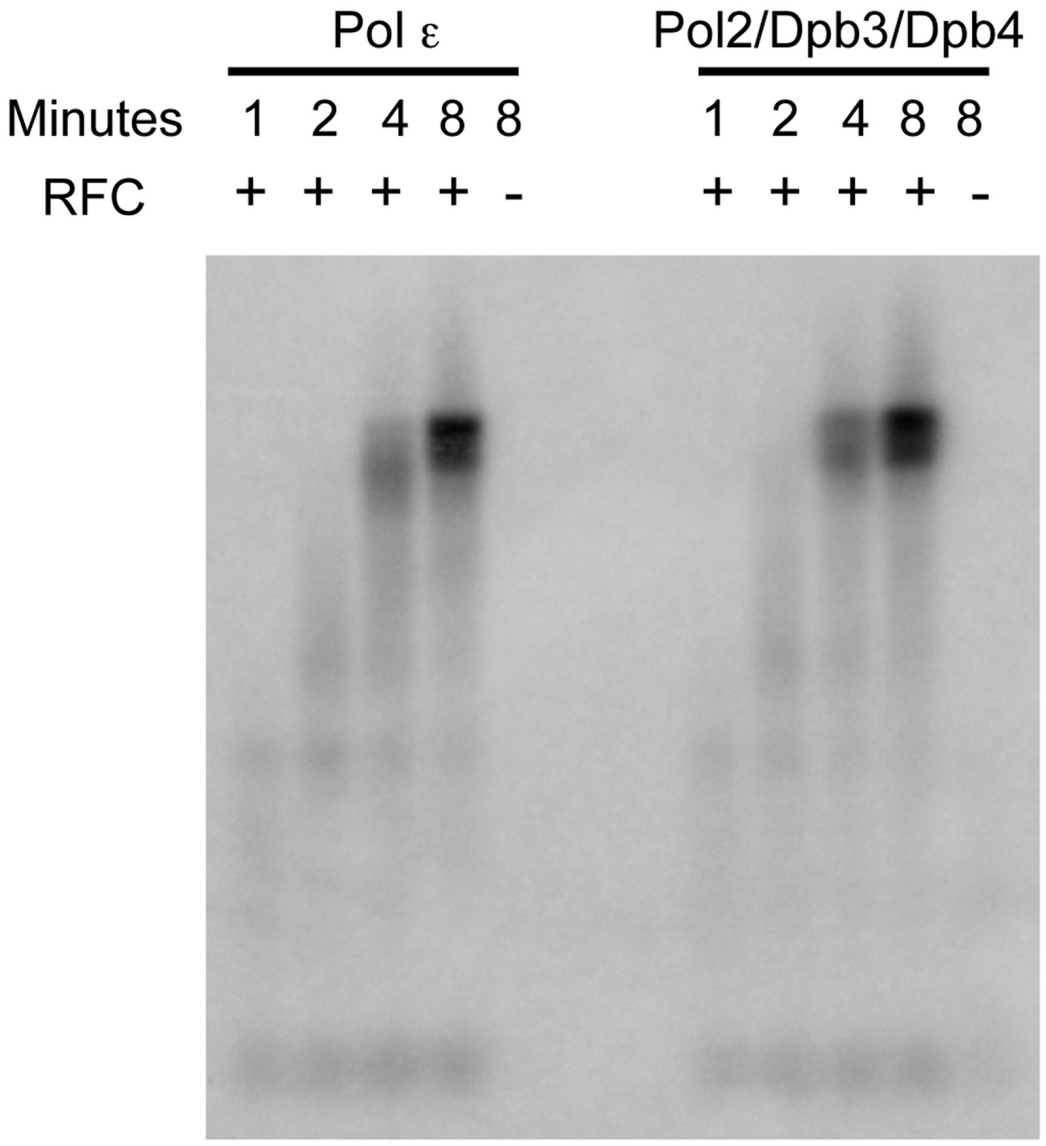
Holoenzyme assay with four-subunit Pol ε and the Pol2/Dpb3/Dpb4 complex. A single-primed pBluescript II SK(+) template was replicated by the indicated Pol ε complex in the presence of RPA and PCNA and in the presence (+) or absence (−) of RFC. Samples were collected after 1, 2, 4 and 8 min.

We used three assays to explore the enzymatic properties of the purified Pol2/Dpb3/Dpb4 complex. The first assay measured the specific activity of the purified Pol2/Dpb3/Dpb4 complex with oligo(dT)-poly(dA) as the substrate. We found that the specific activity of the Pol2/Dpb3/Dpb4 complex was about 2-fold higher than that of the wild-type Pol ε (8860 U/mg and 4551 U/mg, respectively) suggesting that the catalytic activity of the complex was not negatively affected. Processivity is an important parameter for replicative polymerases, and previous studies had shown that Pol ε has a very high intrinsic processivity and that the two non-essential small subunits Dpb3 and Dpb4 are important for this processivity (24). Dpb2 was previously proposed to be located in a structural domain that interacted with the nascent double-stranded DNA and stabilized the interaction with the template (23). To address whether processivity is affected in the Pol2/Dpb3/Dpb4 complex, we performed the previously described experiments that had been performed with the Pol2/Dpb2 complex and wild-type Pol ε (24,40). The reaction conditions at an estimated 1:10 ratio of polymerase to primer were such that the measured termination probability at each position was constant from 2 to 8 min and <20% of the primer was extended. Thus, our experiment met the criteria for single-hit conditions (39). We found that the high processivity of Pol ε in the primer-extension assays was not affected when Dpb2 was missing from the Pol2/Dpb3/Dpb4 complex (Figure 4). There is, however, a strong pause site that limits the analysis to the first 64 nucleotides. This is still a substantial length in comparison to extension by Pol δ that has a processivity of only about five to seven nucleotides in the absence of the processivity clamp (40). Next, we asked whether the loss of Dpb2 affected the interaction of Pol ε with the processivity clamp, PCNA, that stimulates Pol ε in holoenzyme assays. We tested the functional interaction between Pol ε and PCNA on a single-primed circular single-stranded template and found that the loss of Dpb2 did not affect the PCNA-dependent stimulation (Figure 5). Thus, Dpb2 appears not to be essential for the interaction between Pol ε and double-stranded DNA, for achieving high processivity, for loading Pol ε onto the 3′-end of the primer, or for the interaction with PCNA.

## DISCUSSION

In this study, we have identified an essential motif in *S. cerevisiae* Dpb2 that is required for the formation of the four-subunit Pol ε. Earlier studies in *Xenopus laevis*, *S. cerevisiae*, *Schizosaccharomyces pombe* and *Homo sapiens* have shown that the essential C-terminal domain of Pol2 interacts with Dpb2 (41–44). The results from *X. laevis*, *S. cerevisiae* and *H. sapiens* also suggested that the Dpb3/Dpb4 subunits and Dpb2 interact with separate domains of Pol2. Our earlier isolation of a homogenous Pol2/Dpb2 complex (24) and our current results with a Pol2/Dpb3/Dpb4 complex are in agreement with this model.

Pol ε from *S. cerevisiae* was first purified as a 132 kDa proteolytic fragment, and this was later referred to as the 145 kDa fragment (45). This form of Pol ε was missing all accessory subunits because the C-terminal domain where these subunits normally interact with Pol2 was absent. The 145 kDa proteolytic fragment had a potent polymerase activity and, in fact, was reported to have very similar biochemical properties as four-subunit Pol ε (46–48). The differences between the 145 kDa fragment of Pol2 and the Pol ε complex were limited to processivity, salt sensitivity and affinity for single-stranded DNA. Recent studies have focused on the effects of removing the Dpb2, Dpb3 or Dpb4 subunits.

The B subunits of Pol δ (Pol31), Pol α (Pol12) and Pol ε (Dpb2) share conserved motifs, and all are essential in yeast (3). However, their roles remain elusive. Previous studies have shown that Pol31 forms a bridge between Pol3 and Pol32 to improve the efficiency of loading Pol δ onto the PCNA–primer–template ternary complex (12,49). However, this role does not appear to be essential because *POL32* has been shown to be a non-essential gene (11). Recently, it has been shown that the catalytic subunits of both Pol ζ (Rev3) and Pol δ (Pol3) carry an Fe–S cluster at their C-termini (50) and that Pol31 and Pol32 interact with both Pol δ and Pol ζ through the Fe–S cluster (15,50). *DPB2* is an essential gene in both *S. pombe* and *S. cerevisiae* (26,32). However, purified Pol2/Dpb3/Dpb4 complexes from *H. sapiens* were not reported to have any significant changes in their biochemical properties when compared with wild-type Pol ε (42). Our characterization of the Pol2/Dpb3/Dpb4 complex confirms that the loss of Dpb2 does not have a negative influence on Pol ε polymerase activity in the described biochemical assays and results in no loss of protein stability. Moreover, the absence of Dpb2 did not affect the functional interaction between Pol ε and PCNA. Thus, the essential function of Dpb2 appears to be uncoupled from *de novo* DNA synthesis.

Previous studies have suggested that Pol ε contributes to other processes in addition to the synthesis of new DNA. There is strong evidence that Pol ε plays a role in the initiation of DNA replication. In *S. pombe*, a temperature-sensitive *dpb2* mutant was found to be growth-arrested in early S-phase at the non-permissive temperature (32). In addition, Dpb2 was found to be associated with the replication origin in a chromatin precipitation assay (32). In *S. cerevisiae*, a series of experiments have suggested that Pol ε plays a role during the initiation of DNA replication (51), and co-immunoprecipitation experiments have suggested that Dpb2 participates in a pre-loading complex (pre-LC) that consists of Pol ε, GINS, Dpb11 and Sld2 (33). In this complex, both Sld2 and Dpb2 were phosphorylated by CDK. The phosphorylation of Sld2 was shown to be important for regulating the start of DNA replication, but the lack of Dpb2 phosphorylation was not associated with any measurable *in vivo* effects. The role of Pol ε was unclear in the pre-LC, but it positions Pol ε close to the assembly of the CMG helicase complex (Cdc45/Mcm2-7/GINS) (52).

Dpb2 may have an essential function during progression of the replication fork. There is substantial evidence for an asymmetric replication fork in which Pol ε is positioned on the leading strand and Pol δ on the lagging strand (7,8). This raises the question of how this asymmetry is established and maintained. We have previously shown that the processivity clamp, PCNA, was an unlikely candidate for regulating this division of labor because Pol ε and Pol δ are loaded onto the PCNA clamp through different mechanisms (40). In contrast to Pol δ, Pol ε lacks a functional PIP box and we proposed that there might be a PIP box-dependent mechanism that targets Pol δ to the lagging strand and a PIP box-independent mechanism that targets Pol ε to the leading strand (40). The fact that Dpb2 can physically interact with GINS, a part of the CMG complex, suggests that Dpb2 may contribute to targeting Pol ε primarily to the leading strand in DNA synthesis.

In a previous study, a series of temperature-sensitive *dpb2* alleles were isolated. A deficiency in one of the alleles, *dpb2-1*, allowed for the initiation of DNA replication but the progression of the replication fork was perturbed (26). Our current results suggest that the delayed progression of S-phase was unlikely to be due to a defect in the catalytic activity of Pol ε. A caveat with temperature-sensitive alleles, however, is that they fail to properly fold at the non-permissive temperature, and it is possible that mis-folded Dpb2 could influence both the polymerase activity of Pol ε and other protein interactions *in vivo*. Our isolated mutation, *dpb2-200*, was not temperature sensitive, and the deficiency was limited to a local motif at the C-terminus of Dpb2. This makes the deficiency more likely to only affect the interaction with Pol2 and not interactions with other unknown proteins. From that perspective, the essential function of the Pol2 C-terminus might be linked to a physical interaction with Dpb2. It was shown previously that ts-*dpb2* alleles were associated with a significantly increased mutation rate (27,29). It is not clear, however, whether or not the Pol2/Dpb3/Dpb4 complexes, or the Pol2/ts-Dpb2/Dpb3/Dpb4 complexes themselves exhibit reduced fidelity during DNA synthesis compared with the wild-type four-subunit Pol ε. It was recently shown that in some cases, the increased mutation rates observed in ts–*dpb2*-containing strains were a manifestation of a perturbed replication fork (28).

The biological role of Dpb2 remains uncertain. However, we have shown that the C-terminus of Dpb2 is required for its interaction with Pol2 and that this same motif is essential for cell viability. We have also confirmed previous reports that have shown that Dpb2 is not required to support Pol ε polymerase activity in the presence of the accessory proteins PCNA, RFC and RPA. Altogether, the accumulated evidence suggests that Dpb2 plays an essential structural role that remains to be elucidated. Studies are under way to determine the function of Dpb2 with the help of the identified C-terminal motif.

## SUPPLEMENTARY DATA

Supplementary Data are available at NAR Online: Supplementary Figures 1–3.

## FUNDING

Swedish Research Council (to E.J.); Cancerfonden (to E.J.); Smärtafonden (to K.V. and E.J.); Kempe foundation (to I.I., K.V. and E.J.) and Umeå University (to I.I. and E.J.). Funding for open access charge: The Swedish Research Council.

*Conflict of interest statement*. None declared.

## Supplementary Material

Supplementary Data
