# Supplementary material for: The C-terminus of Dpb2 is required for interaction with Pol2 and for cell viability
Source: Nucleic Acids Res. 2012 Oct 2;40(22):11545–53. doi: 10.1093/nar/gks880 (PMC3526264; doi:10.1093/nar/gks880)
Supplement: Supplementary Data [file supp_40_22_11545__index.html]

The C-terminus of Dpb2 is required for interaction with Pol2 and for cell viability — The C-terminus of Dpb2 is required for interaction with Pol2 and for cell viability — Supplementary Data 

# The C-terminus of Dpb2 is required for interaction with Pol2 and for cell viability

## Supplementary Data

files

**Files in this Data Supplement:**

- Supplementary Data - pdf file
