# Supplementary material for: The C-terminus of Dpb2 is required for interaction with Pol2 and for cell viability
Source: Nucleic Acids Res. 2012 Oct 2;40(22):11545–53. doi: 10.1093/nar/gks880 (PMC3526264; doi:10.1093/nar/gks880)

Supplementary figure legends.

Supplemental figure 1: PCR test for deletion of *DPB2* in the E134-*dpb2Δ* strain. Primers KanMXChromDpb2-5 and KanMXChromDpb2-3 that annealed to chromosomal DNA upstream and downstream of *DPB2*, respectively, were used to amplify genomic DNA from A) the E134-*dpb2Δ* strain carrying the pRS316-DPB2 plasmid and B) the E134 strain.

Supplementary figure 2: A) Map describing the integration of *DPB2* or *dpb2-200* with selection for the *hph*-gene. The map is not drawn to scale. Primers used for PCR reactions are named and positioned where they anneal on the chromosome. The expected sizes of PCR-products when correctly integrated are indicated for each primer-pair. B) Agarose gel showing the PCR products when amplified from a strain with the integrated *hph* gene.

Supplementary figure 3: The *hph* gene does not affect cell viability due to its integration 3′ of the wild-type *DPB2* gene. Two independent diploid E134 strains (1A and 10A) heterozygotic for the integration of the *hph* gene next to the *DPB2* gene were sporulated. Three asci from isolate 1A and four asci from isolate 10A were dissected. All four spores were viable from each tested asci (upper panels). The colonies formed by each spore, called A, B, C, and D, were patched on a YPD plate (lower panels). After incubation for two days at 30 °C, they were replica plated onto a YPD plate with 200 μg/mL Hygromycin B. Only the cells carrying the *hph* gene were viable.

Supplemental figure 1

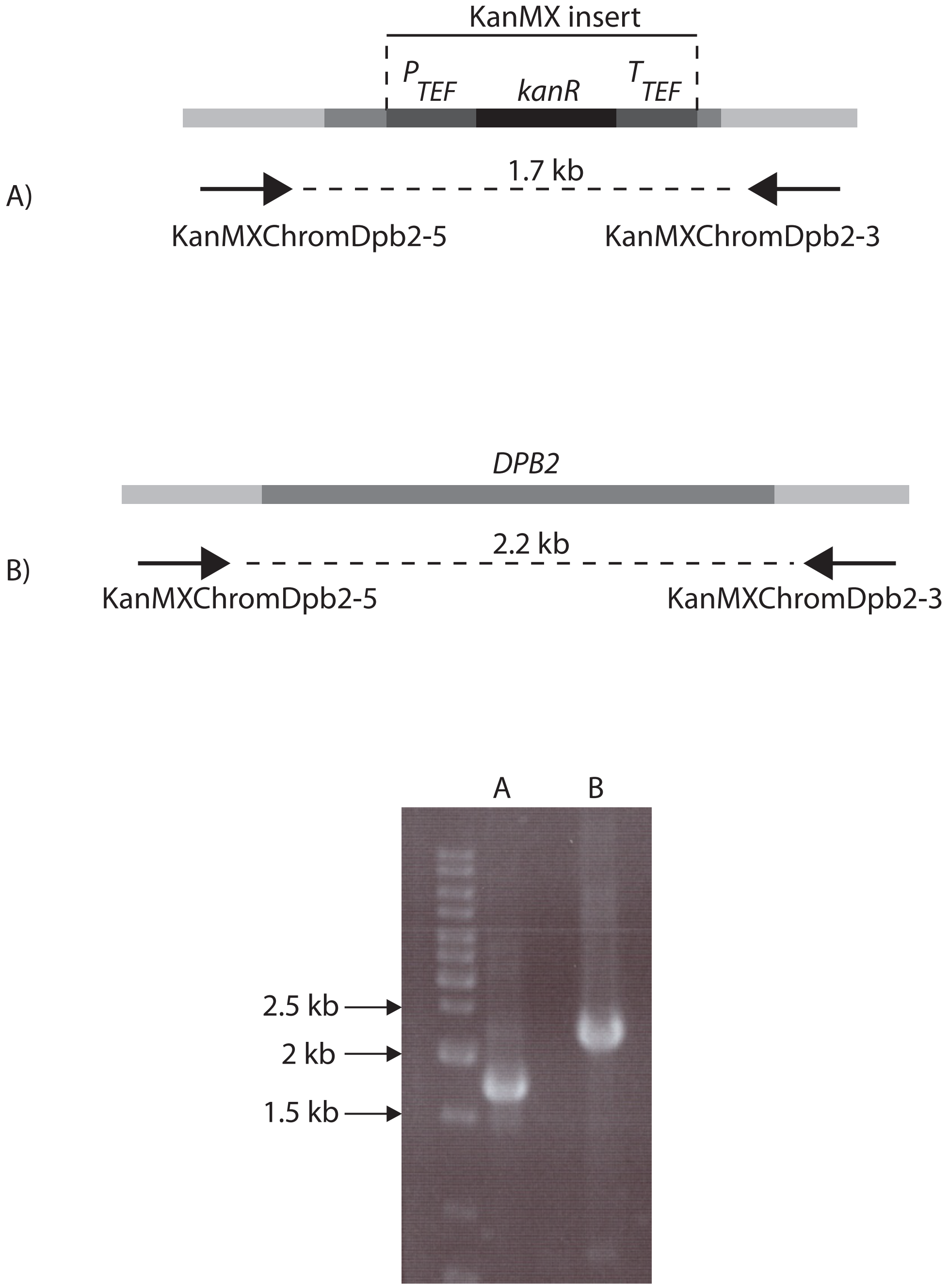

Supplemental figure 2

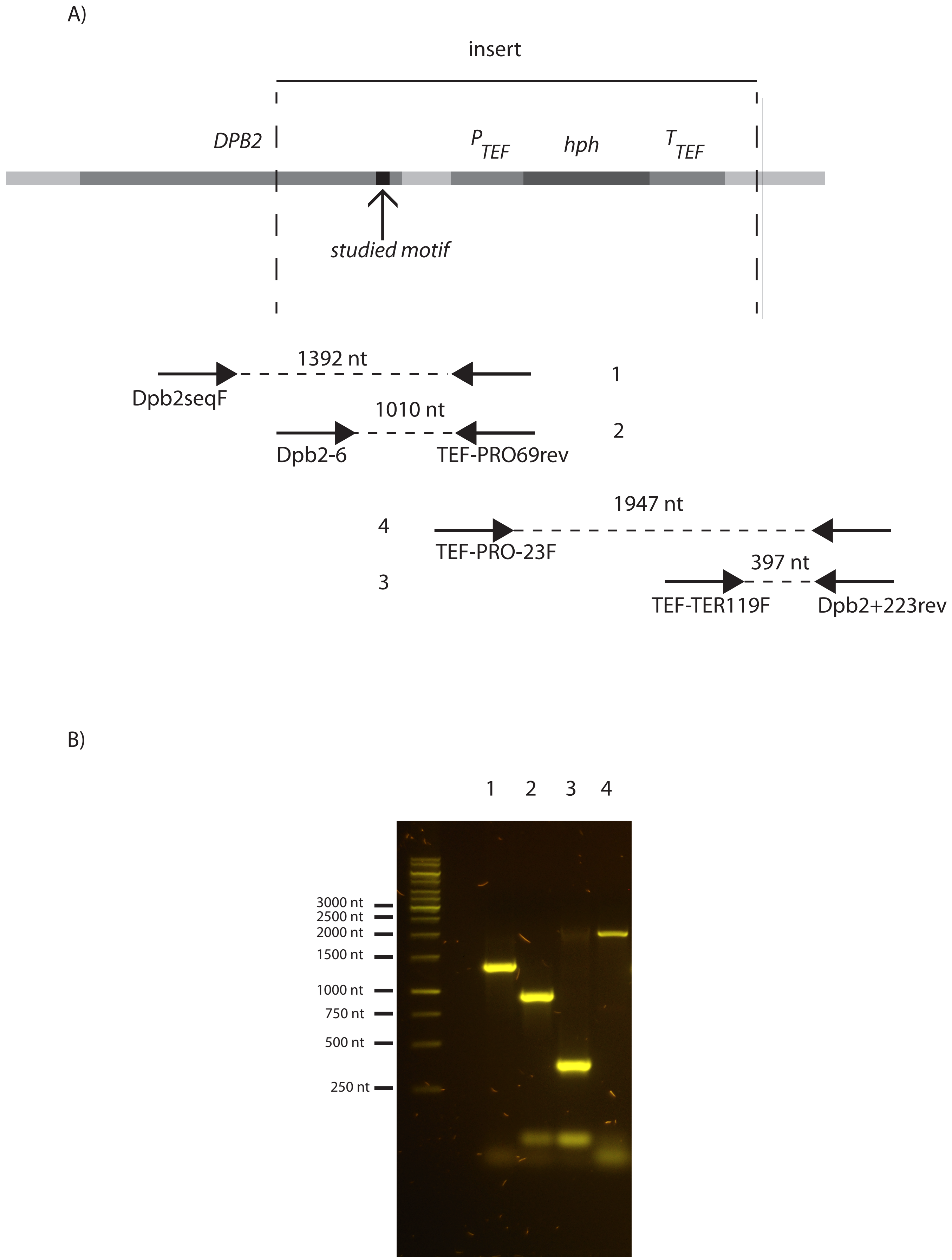

Supplemental figure 3

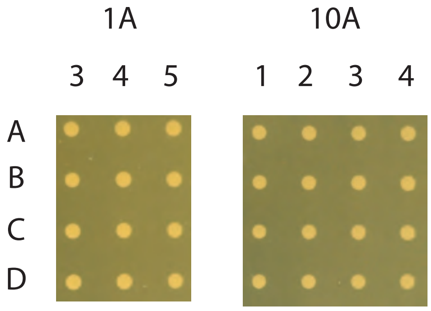

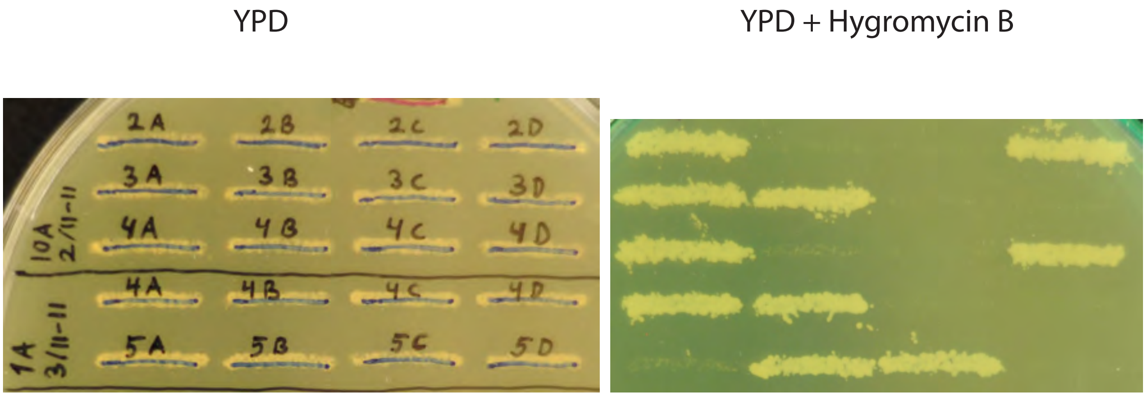

Supplement: Supplementary Data [file supp_gks880_nar-02524-v-2011-File007.pdf]
